# *LINC00682* inhibits gastric cancer cell progression via targeting *microRNA-9*-LMX1A signaling axis

**DOI:** 10.18632/aging.102533

**Published:** 2019-12-11

**Authors:** Xiaohong Zhang, Jian Li, Fan Li, Zhen Zhao, Li Feng

**Affiliations:** 1 Endoscopy Center, Minhang Hospital, Fudan University, Shanghai, China; 2 Department of Clinical Laboratory, Minhang Hospital, Fudan University, Shanghai, China

**Keywords:** gastric cancer, *LINC00682*, *microRNA-9*, LMX1A

## Abstract

*microRNA-9* (“*miR-9*”), upregulated in human gastric cancer (GC) tissues, targets LMX1A (LIM homeobox transcription factor 1α) to promote GC cell progression. The underlying mechanism of *miR-9* upregulation in GC is still unknown. Through searching multiple *long non-coding RNA* (*LncRNA*) databases, we here discovered that the *long non-coding RNA* *LINC00682* (*long intergenic non-protein coding RNA 682*) putatively targets *miR-9*. We show that ectopic overexpression of *LINC00682* induced *miR-9* downregulation but LMX1A upregulation, inhibiting AGS cell survival, proliferation, migration and invasion. Significant apoptosis activation was detected in *LINC00682*-overexpressed AGS cells. Contrarily, *LINC00682* knockdown induced *miR-9* upregulation but LMX1A downregulation, promoting AGS cell survival, proliferation, migration and invasion. In the primary human GC cells, forced *LINC00682* overexpression similarly induced *miR-9* downregulation and LMX1A upregulation, causing proliferation inhibition and apoptosis activation. Significantly, restoring *miR-9* expression by a lentiviral construct reversed *LINC00682*-induced actions in GC cells. Furthermore, *LINC00682* was ineffective in LMX1A KO AGS cells. Importantly, *LINC00682* expression levels are significantly downregulated in human GC tissues. We conclude that *LINC00682* inhibits GC cell progression via targeting *miR-9*-LMX1A signaling axis.

## INTRODUCTION

Gastric cancer (GC) accounts for over 10% of new cancer cases each year [1–3]. It is one leading cause of cancer-associated human mortalities [2, 3]. The prognosis of advanced, recurrent and metastatic GC is far from satisfactory. Current treatment options for this devastating disease are extremely limited [2, 3]. Molecularly-targeted therapies are the research focus for better GC therapies [4, 5]. Identification of novel therapeutic targets and biomarkers of GC is extremely urgent [4, 5].

LIM homeobox transcription factor 1, alpha (LMX1A) is a widely-studied member of LIM-homeodomain (LIM-HD) family protein [6]. As an evolutionary conserved transcription factor, LMX1A regulates a number of key physiological and pathological processes [6]. LMX1A functions as a tumor suppressor, downregulated in GC and many other cancers [7–13]. *LMX1A* gene promoter hyper-methylation in human cancers could be one important cause of its downregulation [7, 8, 10]. Our previous study has identified the LMX1A-targeting *miRNA*, *microRNA-9* (*miR-9*) [9]. *miR-9* upregulation might account for LMX1A downregulation in human GC tissues [9]. Furthermore, *miR-9* inhibition upregulated its target LMX1A, thereby inhibiting GC cell survival and proliferation [9]. The underlying mechanism of *miR-9* upregulation in human GC is still largely unknown.

*Long non-coding RNAs* (*LncRNAs*) are a family of evolutionarily conserved *non-coding RNAs* (*ncRNAs*) with over 200 nucleotide long [14–16]. Dysregulation of *LncRNAs* is commonly detected in GC [17, 18], which is associated with cancer progression and patients’ prognosis [14, 17–20]. *LncRNAs* regulate almost all important cellular functions, from genomic imprinting, cell proliferation and growth, cell cycle progression to cell differentiation, survival and apoptosis [14–16]. *LncRNA* acts as *competing endogenous RNA* (*ceRNA*) to sponge target *miRNAs* [17, 21]. The results of the present study will show that *LINC00682* (*long intergenic non-protein coding RNA 682*) targets *miR-9*-LMX1A signaling axis to inhibit human GC cell survival and proliferation.

## RESULTS

### Ectopic overexpression of *LINC00682* induces *miR-9* downregulation but LMX1A upregulation, inhibiting AGS cell survival, proliferation, migration and invasion

We hypothesized that *miR-9* upregulation in GC tissues (see our previous study [9]) could possibly be due to downregulation of certain *LncRNAs*. Therefore, LncBase (Predicted v.2) was searched to find possible *miR-9-*targeting *LncRNAs*. The *LncRNAs* were further verified by searching other *LncRNA*/*miRNA* databases (StarBase and miRbase). The bioinformatic analyses identified that one *LncRNA*, *LINC00682,* putatively targets *miR-9*, with its percentage over 99%.

**Figure 1:**
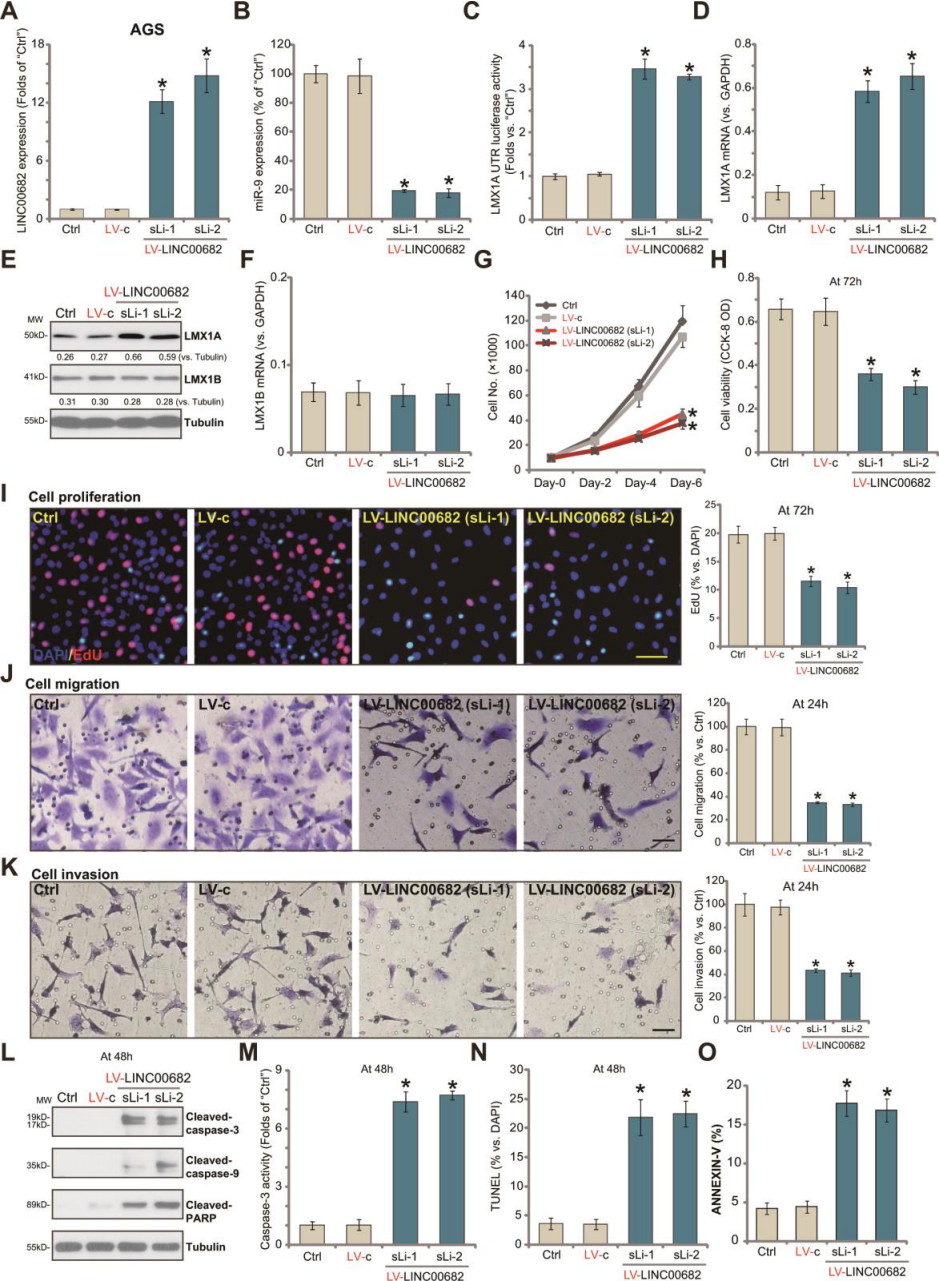
**Ectopic overexpression of *LINC00682* induces *miR-9* downregulation but LMX1A upregulation, inhibiting AGS cell survival, proliferation, migration and invasion.** AGS cells were infected with *LINC00682*-expressing lentivirus (“LV-LINC00682”), following puromycin selection two stable lines (“sLi-1/-2”) were obtained; Control cells were infected with the lentivirus with empty vector (“LV-c”); In those cells expression of *LINC00682* (**A**), *miR-9* (**B**), *LMX1A mRNA* (**D**), *LMX1B mRNA* (**F**) was tested by qPCR; The relative *LMX1A* *3’-UTR* luciferase activity was tested (**C**); Expression of the listed proteins in total cell lysates was tested by Western blotting (**E**); Cells were further cultured for the indicated time periods, cell survival, proliferation, migration and invasion *in vitro* were tested by the appropriate assays (**G**–**K**); Cell apoptosis was tested by Western blotting assay of apoptosis proteins (**L**), caspase-3 activity assay (**M**), nuclear TUNEL staining assay (**N**) and Annexin V FACS staining (**O**). The exact same number of viable cells of different genetic treatments were plated initially (“0h”/“Day-0”) for the functional assays (Same for all following Figures). Five repeated views in each condition were included to calculate the average number of migrated/invasive cells (Same for all Figures). Listed proteins were quantified and normalized to the loading control (**E**). “MW” stands for molecular weight (Same for all Figures). “Ctrl” stands for the parental control cells (Same for all Figures). For each assay, n=5 (five dishes or wells). *P <0.05 *vs.* “LV-c” cells. Experiments in this figure were repeated four times, and similar results were obtained. Bar=100 μm (**I**, **J** and **K**).

In order to study the potential effect of *LINC00682* on *miR-9*-LMX1A axis, the lentivirus encoding *LINC00682*-expressing construct (“LV-LINC00682”) was added to AGS cells. Following selection by the puromycin-containing complete medium, two stable cell lines, “sLi-1” and “sLi-2”, were established. qPCR results confirmed that *LINC00682* levels increased over ten folds (versus control cells) in the LV-LINC00682-expressing stable cells (Figure 1A). Importantly, *LINC00682* overexpression in AGS cells induced significant downregulation of *miR-9* (Figure 1B), but a significant increase in *LMX1A* *UTR* luciferase activity (Figure 1C). Consequently, *LMX1A mRNA* levels increased over five-six folds by LV-LINC00682 (Figure 1D). Western blotting results confirmed that forced overexpression of *LINC00682* induced LMX1A protein upregulation as well (Figure 1E). *LMX1B mRNA* and protein expression was however not significantly affected by LV-LINC00682 (Figure 1E and 1F).

Our previous study has demonstrated that LMX1A functions as a tumor suppressor, inhibiting GC cell survival and proliferation [9]. By counting cell number, we show that forced overexpression of *LINC00682* by LV-LINC00682 significantly inhibited AGS cell growth (Figure 1G). Furthermore, AGS cells with LV-LINC00682 presented with decreased cell viability (CCK-8 OD, Figure 1H) and inhibited EdU ratio (Figure 1I), suggesting proliferation inhibition. Testing cell migration, by the “Transwell” assays, show that LV-LINC00682-induced *LINC00682* overexpression significantly inhibited AGS cell migration *in vitro* (Figure 1J). Furthermore, the “Matrigel Transwell” assay results demonstrated that AGS cell invasion was also suppressed by ectopic *LINC00682* overexpression (Figure 1K).

Importantly, significant apoptosis activation was detected in *LINC00682*-overexpressed AGS cells, evidenced by cleavages of caspase-3, caspase-9 and poly (ADP-ribose) polymerase (PARP) (Figure 1L), as well as increased caspase-3 activation (Figure 1M) and nuclear TUNEL ratio (Figure 1N). Additionally, *LINC00682*-overexpressed AGS cells presented with increased Annexin V staining (Figure 1O). The control lentivirus with empty vector (“LV-c”) had no significant effect on *LINC00682*-*miR-9*-LMX1A/B expression (Figure 1A–1F) nor AGS cell functions (Figure 1G–1O). These results show that ectopic overexpression of *LINC00682* induces *miR-9* downregulation but LMX1A upregulation, inhibiting AGS cell survival, proliferation, migration and invasion.

### *LINC00682* knockdown induces *miR-9* upregulation but LMX1A downregulation, promoting AGS cell survival, proliferation, migration and invasion

**Figure 2:**
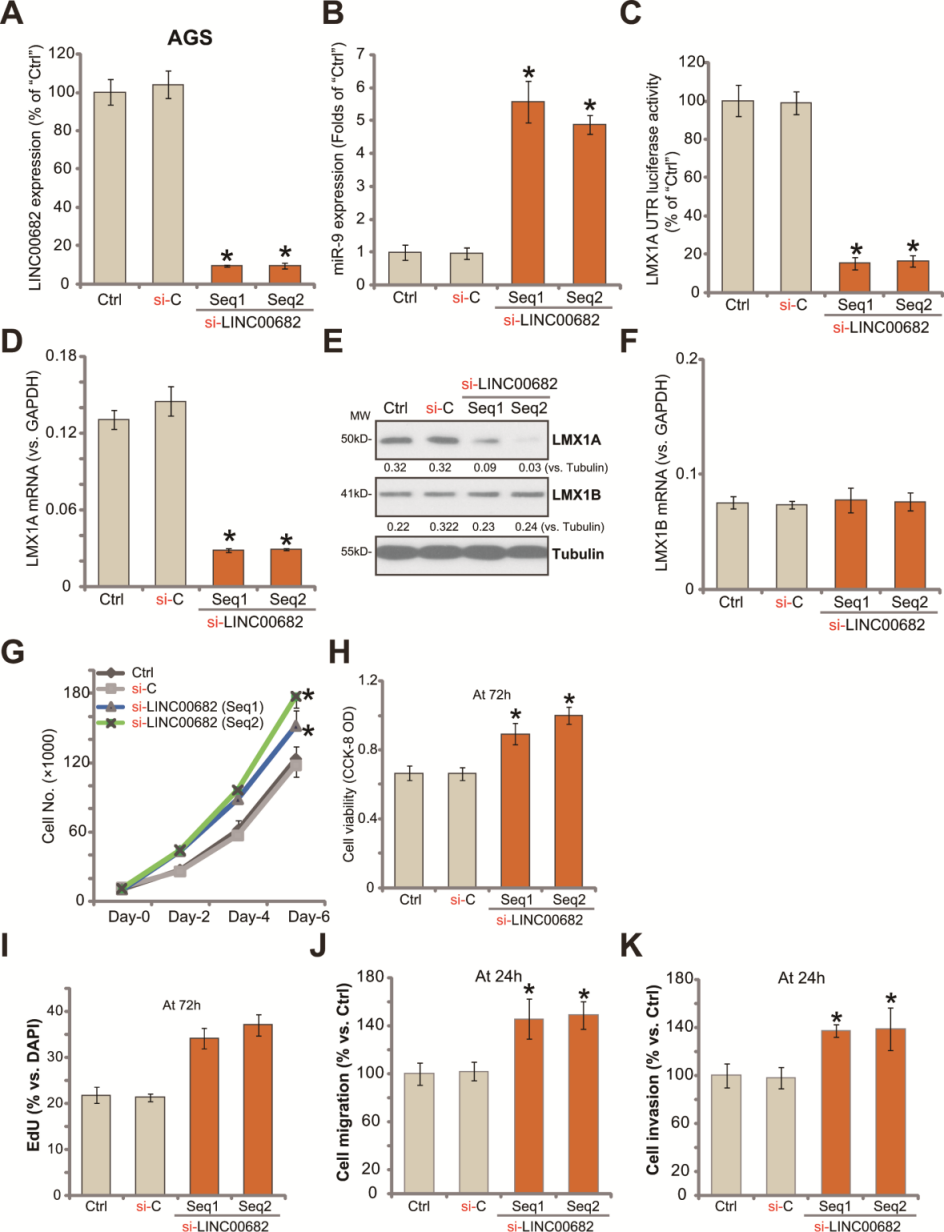
***LINC00682* knockdown induces *miR-9* upregulation but LMX1A downregulation, promoting AGS cell survival, proliferation, migration and invasion.** AGS cells were transfected with 500 nM of *LINC00682* siRNA (“Seq1/Seq2”, two rounds, total 48h) or the scramble non-sense control siRNA (“si-C”), expression levels of *LINC00682* (**A**), *miR-9* (**B**), *LMX1A mRNA* (**D**), *LMX1B mRNA* (**F**) were tested by qPCR; The relative *LMX1A* *3’-UTR* luciferase activity was tested (**C**); Expression of the listed proteins in total cell lysates was tested by Western blotting (**E**); Cells were further cultured for the indicated time periods, cell survival and proliferation *in vitro* were tested by the appropriate assays (G-I); Cell migration and invasion were tested by “Transwell” (J) and “Matrigel Transwell” (K) assays, respectively. Listed proteins were quantified and normalized to the loading control (E). For each assay, n=5 (five dishes or wells). *P <0.05 *vs.* “si-C” cells. Experiments in this figure were repeated four times, and similar results were obtained.

Since exogenous *LINC00682* overexpression inhibited AGS cell progression *in vitro* (Figure 1), we hypothesized that *LINC00682* silencing might promote cell progression. To test this hypothesis, two different siRNAs, targeting non-overlapping sequences (“Seq1/Seq2”) of *LINC00682* were transfected individually to AGS cells. Results from the qPCR confirmed that each siRNA resulted in over 90% reduction of *LINC00682* expression in AGS cells (Figure 2A). *miR-9* levels were significantly increased in *LINC00682*-silenced cells (Figure 2B), where *LMX1A 3’-UTR* luciferase activity was largely decreased (Figure 2C). In AGS cells *LMX1A mRNA* (Figure 2D) and protein (Figure 2E) levels were significantly downregulated by *LINC00682* siRNAs. While the two had no effect on LMX1B expression (Figure 2E and 2F).

For the functional studies, *LINC00682* silencing increased AGS cell growth (Figure 2G), cell viability (CCK-8 OD, Figure 2H), nuclear EdU staining (Figure 2I). Results from “Transwell” and “Matrigel Transwell” assays demonstrated that *LINC00682* silencing by targeted siRNAs promoted AGS cell migration (Figure 2J) and invasion (Figure 2K) *in vitro*. The non-sense scramble control siRNA (“si-C”) had no detectable effect on *LINC00682*-*miR-9*-LMX1A/B expression (Figure 2A–2F) nor AGS cell functions (Figure 2G–2K). Therefore, *LINC00682* knockdown promoted AGS cell progression *in vitro*.

### Ectopic overexpression of *LINC00682* induces *miR-9* downregulation but LMX1A upregulation, inhibiting survival and proliferation of primary human GC cells

**Figure 3:**
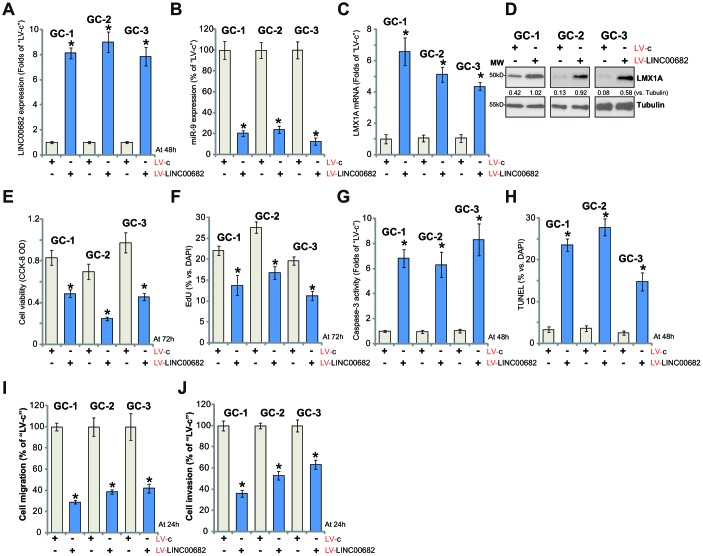
**Ectopic overexpression of *LINC00682* induces *miR-9* downregulation but LMX1A upregulation, inhibiting survival and proliferation of primary human GC cells.** The primary human GC cells, derived from three different primary GC patients (“GC-1/GC-2/GC-3”), were infected with *LINC00682*-expressing lentivirus (“LV-LINC00682”) or the lentivirus with empty vector (“LV-c”), followed by puromycin selection; Expression levels of *LINC00682* (**A**), *miR-9* (**B**), *LMX1A mRNA* (**C**), and listed proteins (**D**) were tested; Cells were further cultured for the indicated time periods, cell viability (CCK-8 OD, **E**) and proliferation (EdU staining, **F**) were tested; Cell apoptosis was tested by caspase-3 activity assay (**G**) and TUNEL staining (**H**). Cell migration and invasion were tested by “Transwell” (**I**) and “Matrigel Transwell” (**J**) assays, respectively. Listed proteins were quantified and normalized to the loading control (**D**). For each assay, n=5 (five dishes or wells). *P <0.05 *vs.* “LV-c” cells. Experiments in this figure were repeated three times, and similar results were obtained.

The potential effect of *LINC00682* on the primary GC cells was studied next. As reported [9], the primary human GC cells were derived from three different primary GC patients (“GC-1/GC-2/GC-3”) and cultured *in vitro*. The primary cells were infected with LV-LINC00682 (see Figure 1), followed by selection in puromycin-containing medium. Control cells were treated with the control lentivirus (“LV-c”, see Figure 1). qPCR results in Figure 3A confirmed that *LINC00682* levels were significantly increased (over eight to ten folds) in stable primary cancer cells with LV-LINC00682. Forced overexpression of *LINC00682* induced *miR-9* downregulation (Figure 3B), while upregulating *LMX1A mRNA* (Figure 3C) and protein (Figure 3D) in the primary GC cells.

When studying cellular functions, we show that LV-LINC00682 inhibited cell viability (Figure 3E) and EdU staining (Figure 3F) in primary GC cells. On the other hand, cell apoptosis, tested by caspase-3 activation (Figure 3G) and TUNEL ratio increase (Figure 3H), was induced by LV-LINC00682. “Transwell” and “Matrigel Transwell” assay results, Figure 3I and 3J, demonstrated that ectopic *LINC00682* overexpression inhibited *in vitro* migration and invasion of the primary human GC cells. These results show that *LINC00682* overexpression inhibited primary GC cell survival and proliferation, migration and invasion, while provoking apoptosis activation.

### *LINC00682* inhibits AGS cell progression via targeting *miR-9*-LMX1A axis

**Figure 4:**
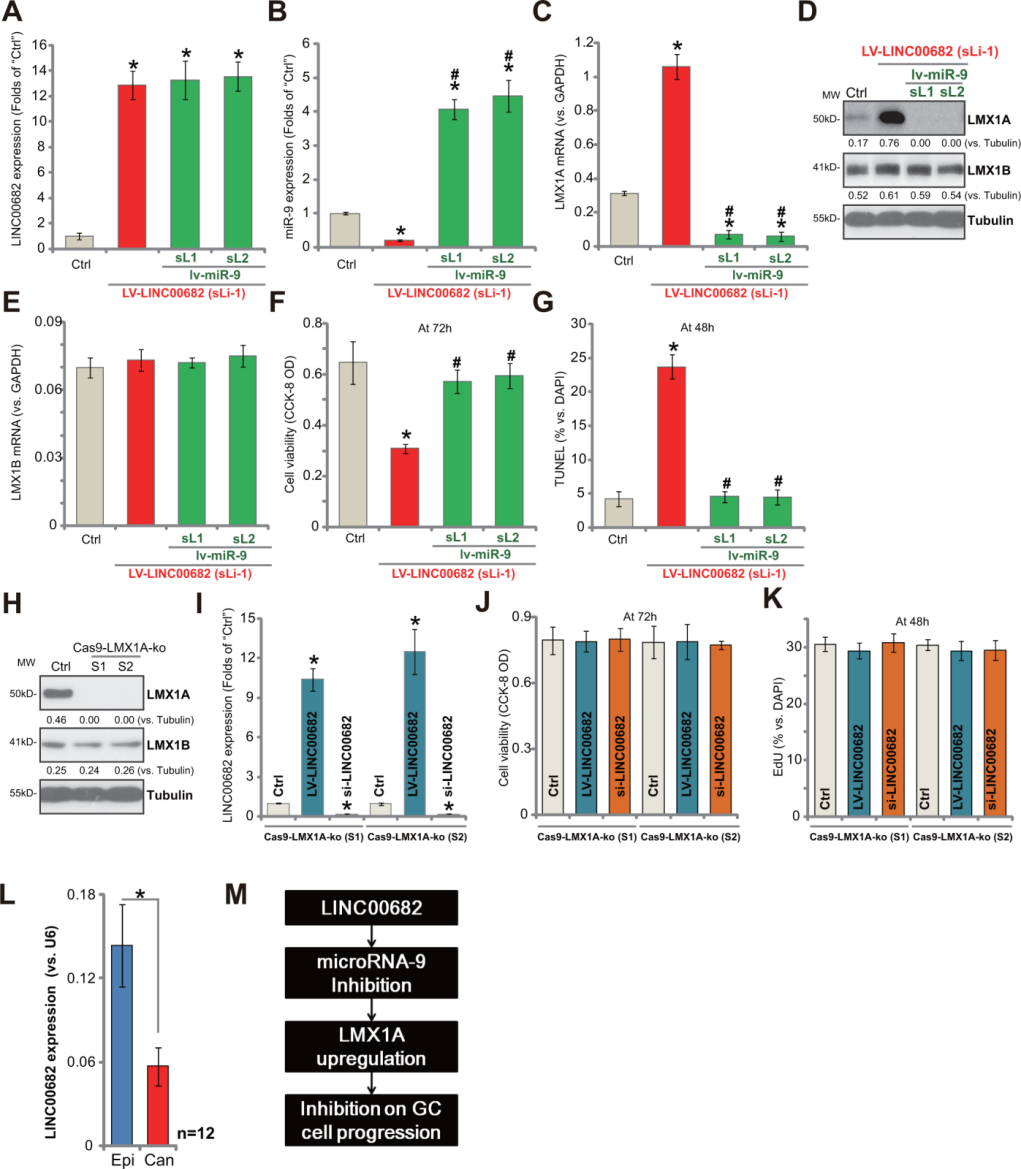
***LINC00682* inhibits AGS cell progression via targeting *miR-9*-LMX1A axis.** AGS cells were infected with *LINC00682*-expressing lentivirus (“LV-LINC00682”), following puromycin selection the stable cells were established. The stable cells (“sLi-1”) were further infected with *pri-miR-9*-expressing lentivirus (“lv-miR-9”) for 24h, following puromycin selection two stable lines were obtained (“sL1/ sL2”); In the cells expression of *LINC00682* (**A**), *miR-9* (**B**), *LMX1A mRNA* (**C**), listed proteins (**D**) and *LMX1B mRNA* (E) was tested; Cells were further cultured for applied time, and cell viability (**F**) and apoptosis (**G**) were tested by the appropriate assays. AGS cells were transfected with the lenti-CRISPR/Cas9 LMX1A knockout constructs with non-overlapping sgRNA sequences (“S1/S2”), following FACS sorting and puromycin selection two stable lines were obtained (“Cas9-LMX1A-ko”). LMX1A and LMX1B expression was tested (**H**). LV-LINC00682 or *LINC00682* siRNA (500 nM) were transfected to the Cas9-LMX1A-ko AGS cells (“S1/S2”) for 72h, *LINC00682* expression (**I**), cell viability (**J**) and proliferation (**K**) were tested. Expression of *LINC00682* in twelve (n=12) human GC tissues (“Can”) and matched surrounding normal epithelial tissues (“Epi”) was tested by qPCR, and results were normalized to *U6 RNA* (**L**). The proposed signaling pathway of this study (**M**). Listed proteins were quantified and normalized to the loading control (**D** and **H**). For each assay, n=5 (five dishes or wells, except for **L**). *P <0.05 *vs.* “Ctrl” cells. ^#^ *P* <0.05 *vs.* cells without “lv-miR-9” (**B**, **C**, **E**–**G**). *P <0.05 (**L**). Experiments in this figure were repeated three times, and similar results were obtained.

If *miR-9* is the primary target of *LINC00682*, restoring *miR-9* expression should abolish *LINC00682*-induced actions in GC cells. Thus, in the stable AGS cells with LV-LINC00682 (“sLi-1”, see Figure 1), the *miR-9*-expressing lentivirus (“lv-miR-9”, see our previous study [9]) was added. Two stable cell lines were established, “sL1/sL2”. As shown, in LV-LINC00682 AGS cells, lv-miR-9 did not affect *LINC00682* expression (Figure 4A). Yet it restored *miR-9* expression, four-five times higher to the control level (Figure 4B). Further, LV-LINC00682-induced *LMX1A mRNA* (Figure 4C) and protein (Figure 4D) upregulation was completely blocked by lv-miR-9. *LMX1B mRNA* was again unchanged (Figure 4E). Importantly, LV-LINC00682-induced viability (CCK-8 OD) reduction (Figure 4F) and apoptosis activation (the increase in TUNEL staining) (Figure 4G) were abolished by lv-miR-9 in AGS cells. These results showed that ectopic *miR-9* expression reversed *LINC00682*-induced inhibition on GC cells, suggesting that *miR-9* is the target of *LINC00682*.

Our study has previously shown that LMX1A is the direct and primary target of *miR-9* in GC cells, therefore *LINC00682* should be ineffective in LMX1A-depleted cells. To test this hypothesis, the CRISPR/Cas9 method by using non-overlapping sgRNA sequences (“S1/S2”) [9] was utilized to knockout LMX1A in AGS cells. Two stable LMX1A knockout (“Cas9-LMX1A-ko”) cell lines were established. Testing LMX1A protein expression by Western blotting confirmed complete LMX1A KO in the stable cells (Figure 4H). LV-LINC00682 (see Figure 1) or LINC00682 siRNA (“Seq1”, see Figure 2) were transfected to LMX1A KO AGS cells, which significantly altered *LINC00682* expression (Figure 4I). Yet, neither LV-LINC00682 nor LINC00682 siRNA affected viability (CCK-8 OD, Figure 4J) and proliferation (EdU ratio, Figure 4K) in the LMX1A KO cells. Therefore, *LINC00682* was completely ineffective in LMX1A KO AGS cells, confirming LMX1A is the target protein of *LINC00682*.

### *LINC00682* is downregulated in human GC tissues

Expression of *LINC00682* in human GC tissues was tested. Total RNA was extracted from fresh GC tissues and paired adjacent normal epithelial tissues from twelve (12) primary GC patients [9]. *LINC00682* expression was examined by qPCR. Results show that *LINC00682* levels are significantly downregulated in cancer tissues (“Can”) (Figure 4L), when compared to those in the adjacent epithelial (“Epi”) tissues (Figure 4L). Therefore, *LINC00682* downregulation correlates with *miR-9* upregulation and LMX1A downregulation in GC tissues (see the results from same set of tissue samples [9]).

## DISCUSSION

LMX1A is hyper-methylated and downregulated in ovarian cancer and cervical cancer [12, 13]. Recent studies have proposed LMX1A as a tumor suppressor in GC and other cancers. Chao et al., have shown that LMX1A can inhibit tumorigenesis, epithelial-mesenchymal transition (EMT) and stem-like properties of epithelial ovarian cancer [11]. Its expression is associated with advanced stages, poor differentiation, early recurrence and poor overall survival in ovarian cancer [11]. Feng et al., demonstrated that LMX1A inhibited GC cell metastasis through negative regulation of β-catenin [10]. Our previous study has demonstrated that LMX1A is downregulated in human GC. Furthermore CRISPR/Cas9-mediated LMX1A KO promoted GC cell proliferation [9]. Conversely, LMX1A upregulation by *miR-9* depletion led to GC cell apoptosis [9]. However, the regulation of LMX1A in carcinogenesis remains largely unknown.

Very few studies have explored the potential biological function of *LINC00682*. One genome wide methylation study between primary and recurrent hepatocellular carcinomas (HCC) has indicated that low levels of *LINC00682* methylation were strongly correlated with HCC recurrence and patient disease/recurrent-free survival (DFS) [22]. In the present study, our results indicate that *LINC00682* could function as the *ceRNA* of *miR-9* to promote LMX1A expression, thereby inhibiting GC cell progression.

We show that ectopic overexpression of *LINC00682* induced *miR-9* downregulation but LMX1A upregulation, inhibiting GC cell survival, proliferation migration and invasion. Furthermore, significant apoptosis activation was detected in *LINC00682*-overexpressed GC cells. On the contrary, *LINC00682* knockdown by targeted siRNAs induced *miR-9* upregulation but LMX1A downregulation, promoting AGS cell proliferation, migration and invasion. Significantly, *LINC00682* expression levels are downregulated in human GC tissues, correlated with *miR-9* upregulation and LMX1A downregulation. Thus, *LINC00682* downregulation could be at least one reason of *miR-9*-LMX1A dysregulation in human GC (see proposed signaling carton in Figure 4M).

Our results suggest that *miR-9*-LMX1A signaling axis is the direct target of *LINC00682* in GC cells. In AGS cells, restoring *miR-9* expression by lv-miR-9 reversed LV-LINC00682-induced LMX1A upregulation and cancer cell inhibition. Furthermore, we have previously shown that *miR-9* inhibition by antagomir-9 increased *LMX1A* expression to inhibit GC cell proliferation [9]. Thus, antagomir-9 exerted similar functions as LV-LINC00682. Our results demonstrated that neither *LINC00682* overexpression nor *LINC00682* silencing altered the functions of LMX1A KO AGS cells. We conclude that LMX1A is the target protein of *LINC00682* in GC cells.

In conclusion, *LINC00682* inhibits GC cell progression via targeting *miR-9*-LMX1A signaling axis.

## MATERIALS AND METHODS

### Chemicals and reagents

Puromycin and polybrene were provided by Sigma-Aldrich (St. Louis, Mo). The antibodies of the present study were obtained from Abcam (Cambridge, MA). The reagents for RNA assays, Lipofectamine 2000 and other transfection reagents were provided by Thermo-Fisher (Shanghai, China). All sequences, constructs, viruses and plasmids were provided by Shanghai Genechem Co. (Shanghai, China).

### Cell culture

Using a previously described protocol AGS cells were cultured [9]. The primary human GC cells, derived from three written-informed consent GC patients (“GC-1/-2/-3”), were cultured in the described medium [23]. The enrolled primary GC patients in this study received no prior chemotherapy/radiotherapy before surgeries. The protocols were approved by the Ethics Board of Fudan University, in according to Declaration of Helsinki.

### Human tissues

As described early [9], from twelve (12) informed-consent primary GC patients, the fresh human GC tissues and paired surrounding gastric epithelial tissues were acquired. Tissues were washed, minced, and homogenized in tissue lysis buffer (Biyuntian, Wuxi, China), stored in liquid nitrogen. Expression of *LINC00682* was tested by quantitative reverse transcriptase PCR (“qPCR”).

### qPCR assay

The detailed protocol for qPCR was described early [9]. The ^ΔΔ^Ct method was utilized for the quantification of target *mRNA*, with *GAPDH* as the internal control. *LINC00682* and *miR-9* expression was normalized to *U6 RNA*. Primers for *miR-9*, *LMX1A*, *GAPDH* and *U6* were described previously [9]. The primers for *LINC00682* were provided by Shanghai Genechem (Shanghai, China).

### Forced *LINC00682* overexpression

The full-length *LINC00682* was synthesized by Shanghai Genechem, sub-cloned to a GV248 (hU6-MCS-Ubiquitin-EGFP-IRES-puromycin) vector (Shanghai Genechem). The construct was transfected to HEK-293 cells together with lentiviral packaging plasmids [9] to generate *LINC00682*-expressing lentivirus (“LV-LINC00682”). After filtration and enrichment, the lentivirus was added to cultured GC cells (in polybrene medium). Thereafter, puromycin (5.0 μg/mL) was added to select stable cells for 4-5 passages. Control cells were infected with lentivirus with empty vector (“LV-c”).

### *LINC00682* small interfering RNA (siRNA)

Two siRNAs (provided and verified again by Shanghai Genechem) targeting non-overlapping sequences (“Seq1/2”) of *LINC00682* were individually transfected by Lipofectamine 2000 for 24h (the siRNA concentration at 500 nM). The transfection was repeated another round (total 48h). Knockdown efficiency was verified by qPCR testing *LINC00682* expression. Control cells were transfected with the scramble non-sense control siRNA (“si-C”).

### *LMX1A 3’-UTR* luciferase reporter assay

As reported early [9], AGS cells were transfected with pGL4.13 *LMX1A* 3’-UTR construct [9], the Renillaluciferase reporter vector and pRL-SV40 (Promega). In AGS cells following transfection of *LINC00682* siRNA or LV-LINC00682, *LMX1A* *3’-UTR* luciferase activity was tested as reported [9].

### Cell viability

GC cells were seeded into the 96-well plates at 5000 cells per well [9]. Following the applied genetic treatments, the viability was determined by Cell Counting kit-8 (CCK-8, Dojindo Laboratories, Kumamoto, Japan). CCK-8 optical density (OD) values were tested at the wavelength of 570 nm.

### *In vitro* cell migration and invasion assays

As described [24], GC cells were seeded on “Transwell” upper chamber (at 3 × 4000 cells per chamber, BD Biosciences). The complete medium (with 10% FBS) was added to the lower compartments. After 24h the migrated cells on the lower surface were stained. Matrigel (Sigma) was added in the chamber surface when analyzing cell invasion. Five repeated views in each condition were included to calculate the average number of migrated/invasive cells.

### EdU assay

Following the genetic treatments, GC cells were seeded onto 12-well plates at 30, 000 cells per well. The EdU (5-ethynyl-20-deoxyuridine) Apollo-567 Kit (RIBOBIO, Shanghai, China) was employed to quantify cell proliferation. EdU and DAPI dyes were added to GC cells for 6h. Under a fluorescent microscope cell nuclei were visualized. For each condition 800 nuclei in five random views were included to calculate EdU ratio (EdU/DAPI×100%).

### Apoptosis assays

Testing cell apoptosis, by the caspase-3 activity assay, terminal deoxynucleotidyl transferase dUTP nick-end labeling (TUNEL), and Propidium iodide (PI)-Annexin V FACS assay was described in other studies [9, 25].

### LMX1A knockout

AGS cells were seeded into six-well plates at 100, 000 cells per well. The two lentiCRISPR-GFP-puro LMX1A knockout constructs (with non-overlapping small guide RNAs [“S1/S2”], see our previous study [9]) were individually transfected to AGS cells by Lipofectamine 2000. FACS-sorting of GFP-positive cells was performed, and resulting cells were further cultured in puromycin-containing medium. LMX1A knockout in the stable cells was verified by Western blotting.

### Western blotting

Following the genetic treatments, the lysis buffer (Biyuntian, Wuxi, China) was added to cultured GC cells. Quantified total cellular lysates were separated by a SDS-PAGE (10%) gel, transferred to a polyvinylidene fluoride blot. The latter was blocked in PBST with 10% milk, and incubated with indicated primary and secondary antibodies. The immunocomplexes were visualized using an ECL substrate kit (Amersham International, Amersham, UK). Quantification of the target protein bands was through the ImageJ software measuring the total gray. The value was normalized to the loading control.

### Statistical analyses

For statistical analyses the SPSS software (version 18.0) was employed. All values were expressed as the mean ± standard deviation (SD). All differences were considered significant at *P* < 0.05.

## References

[1] Siegel RL, Miller KD, Jemal A. Cancer statistics, 2018. CA Cancer J Clin. 2018; 68:7–30. 10.3322/caac.21442 29313949

[2] Choi KS, Suh M. Screening for gastric cancer: the usefulness of endoscopy. Clin Endosc. 2014; 47:490–96. 10.5946/ce.2014.47.6.490 25505713 PMC4260095

[3] Hashim D, Boffetta P, La Vecchia C, Rota M, Bertuccio P, Malvezzi M, Negri E. The global decrease in cancer mortality: trends and disparities. Ann Oncol. 2016; 27:926–33. 10.1093/annonc/mdw027 26802157

[4] Shah MA. Gastrointestinal cancer: targeted therapies in gastric cancer-the dawn of a new era. Nat Rev Clin Oncol. 2014; 11:10–11. 10.1038/nrclinonc.2013.231 24300880

[5] Wadhwa R, Song S, Lee JS, Yao Y, Wei Q, Ajani JA. Gastric cancer-molecular and clinical dimensions. Nat Rev Clin Oncol. 2013; 10:643–55. 10.1038/nrclinonc.2013.170 24061039 PMC3927982

[6] Doucet-Beaupré H, Ang SL, Lévesque M. Cell fate determination, neuronal maintenance and disease state: the emerging role of transcription factors Lmx1a and Lmx1b. FEBS Lett. 2015; 589:3727–38. 10.1016/j.febslet.2015.10.020 26526610

[7] Dong W, Feng L, Xie Y, Zhang H, Wu Y. Hypermethylation-mediated reduction of LMX1A expression in gastric cancer. Cancer Sci. 2011; 102:361–66. 10.1111/j.1349-7006.2010.01804.x 21159062

[8] Chang CC, Huang RL, Wang HC, Liao YP, Yu MH, Lai HC. High methylation rate of LMX1A, NKX6-1, PAX1, PTPRR, SOX1, and ZNF582 genes in cervical adenocarcinoma. Int J Gynecol Cancer. 2014; 24:201–09. 10.1097/IGC.0000000000000054 24407576

[9] Zhang X, Qian Y, Li F, Bei S, Li M, Feng L. microRNA-9 selectively targets LMX1A to promote gastric cancer cell progression. Biochem Biophys Res Commun. 2018; 505:405–12. 10.1016/j.bbrc.2018.09.101 30262143

[10] Feng L, Xie Y, Zhao Z, Lian W. LMX1A inhibits metastasis of gastric cancer cells through negative regulation of β-catenin. Cell Biol Toxicol. 2016; 32:133–39. 10.1007/s10565-016-9326-0 27061089

[11] Chao TK, Yo YT, Liao YP, Wang YC, Su PH, Huang TS, Lai HC. LIM-homeobox transcription factor 1, alpha (LMX1A) inhibits tumourigenesis, epithelial-mesenchymal transition and stem-like properties of epithelial ovarian cancer. Gynecol Oncol. 2013; 128:475–82. 10.1016/j.ygyno.2012.12.018 23270808

[12] Su HY, Lai HC, Lin YW, Chou YC, Liu CY, Yu MH. An epigenetic marker panel for screening and prognostic prediction of ovarian cancer. Int J Cancer. 2009; 124:387–93. 10.1002/ijc.23957 18942711

[13] Lai HC, Lin YW, Huang TH, Yan P, Huang RL, Wang HC, Liu J, Chan MW, Chu TY, Sun CA, Chang CC, Yu MH. Identification of novel DNA methylation markers in cervical cancer. Int J Cancer. 2008; 123:161–67. 10.1002/ijc.23519 18398837

[14] Niu ZS, Niu XJ, Wang WH. Long non-coding RNAs in hepatocellular carcinoma: potential roles and clinical implications. World J Gastroenterol. 2017; 23:5860–74. 10.3748/wjg.v23.i32.5860 28932078 PMC5583571

[15] Huo X, Han S, Wu G, Latchoumanin O, Zhou G, Hebbard L, George J, Qiao L. Dysregulated long noncoding RNAs (lncRNAs) in hepatocellular carcinoma: implications for tumorigenesis, disease progression, and liver cancer stem cells. Mol Cancer. 2017; 16:165. 10.1186/s12943-017-0734-4 29061150 PMC5651571

[16] Yang X, Xie X, Xiao YF, Xie R, Hu CJ, Tang B, Li BS, Yang SM. The emergence of long non-coding RNAs in the tumorigenesis of hepatocellular carcinoma. Cancer Lett. 2015; 360:119–24. 10.1016/j.canlet.2015.02.035 25721084

[17] Hao NB, He YF, Li XQ, Wang K, Wang RL. The role of miRNA and lncRNA in gastric cancer. Oncotarget. 2017; 8:81572–82. 10.18632/oncotarget.19197 29113415 PMC5655310

[18] Sun M, Nie FQ, Wang ZX, De W. Involvement of lncRNA dysregulation in gastric cancer. Histol Histopathol. 2016; 31:33–39. 26302456 10.14670/HH-11-655

[19] Chen L, Dzakah EE, Shan G. Targetable long non-coding RNAs in cancer treatments. Cancer Lett. 2018; 418:119–24. 10.1016/j.canlet.2018.01.042 29341880

[20] Arun G, Diermeier SD, Spector DL. Therapeutic Targeting of Long Non-Coding RNAs in Cancer. Trends Mol Med. 2018; 24:257–77. 10.1016/j.molmed.2018.01.001 29449148 PMC5840027

[21] Qu S, Yang X, Li X, Wang J, Gao Y, Shang R, Sun W, Dou K, Li H. Circular RNA: A new star of noncoding RNAs. Cancer Lett. 2015; 365:141–48. 10.1016/j.canlet.2015.06.003 26052092

[22] Cui C, Lu Z, Yang L, Gao Y, Liu W, Gu L, Yang C, Wilson J, Zhang Z, Xing B, Deng D, Sun ZS. Genome-wide identification of differential methylation between primary and recurrent hepatocellular carcinomas. Mol Carcinog. 2016; 55:1163–74. 10.1002/mc.22359 26138747

[23] Yang L, Zheng LY, Tian Y, Zhang ZQ, Dong WL, Wang XF, Zhang XY, Cao C. C6 ceramide dramatically enhances docetaxel-induced growth inhibition and apoptosis in cultured breast cancer cells: a mechanism study. Exp Cell Res. 2015; 332:47–59. 10.1016/j.yexcr.2014.12.017 25576381

[24] Wang SS, Lv Y, Xu XC, Zuo Y, Song Y, Wu GP, Lu PH, Zhang ZQ, Chen MB. Triptonide inhibits human nasopharyngeal carcinoma cell growth via disrupting Lnc-RNA THOR-IGF2BP1 signaling. Cancer Lett. 2019; 443:13–24. 10.1016/j.canlet.2018.11.028 30503558

[25] Chen MB, Liu YY, Xing ZY, Zhang ZQ, Jiang Q, Lu PH, Cao C. Itraconazole-Induced Inhibition on Human Esophageal Cancer Cell Growth Requires AMPK Activation. Mol Cancer Ther. 2018; 17:1229–39. 10.1158/1535-7163.MCT-17-1094 29592879

